# Effects of deployment on diet quality and nutritional status markers of elite U.S. Army special operations forces soldiers

**DOI:** 10.1186/s12937-017-0262-5

**Published:** 2017-07-03

**Authors:** Emily K. Farina, Jonathan C. Taylor, Gary E. Means, Nancy E. Murphy, Stefan M. Pasiakos, Harris R. Lieberman, James P. McClung

**Affiliations:** 1 Henry Jackson Foundation for the Advancement of Military Medicine, 6720-A Rockledge Drive, Suite 100, Bethesda, MD 20817 USA; 2 Oak Ridge Institute for Science and Education, 492 Millennium Drive, Suite 101, Belcamp, MD 21017 USA; 3 Department of Health and Human Sciences, Office of the National Coordinator for Health Information Technology, 330 C Street, NW, Washington, DC 20201 USA; 4 U.S. Army Special Operations Command, 2929 Desert Storm Drive, Fort Bragg, NC 28303 USA; 5 U.S. Army Research Institute of Environmental Medicine, Military Nutrition Division, 10 General Greene Avenue, Building 42, Natick, MA 01760 USA

**Keywords:** Healthy eating index, Iron, Ferritin, Calcium, Vitamin D, Parathyroid hormone, Military

## Abstract

**Background:**

Special Operations Forces (SOF) Soldiers deploy frequently and require high levels of physical and cognitive performance. Nutritional status is linked to cognitive and physical performance. Studies evaluating dietary intake and nutritional status in deployed environments are lacking. Therefore, this study assessed the effects of combat deployment on diet quality and serum concentrations of nutritional status markers, including iron, vitamin D, parathyroid hormone (PTH), glucose, and lipids, among elite United States (U.S.) Army SOF Soldiers.

**Methods:**

Changes from baseline to post-deployment were determined with a repeated measure within-subjects design for Healthy Eating Index-2010 (HEI-2010) scores, intake of foods, food groups, key nutrients, and serum nutritional status markers. Dietary intake was assessed with a Block Food Frequency Questionnaire. The association between post-deployment serum 25-hydroxy vitamin D (25-OH vitamin D) and PTH was determined. Analyses of serum markers were completed on 50 participants and analyses of dietary intake were completed on 33 participants.

**Results:**

In response to deployment, HEI-2010 scores decreased for total HEI-2010 (70.3 ± 9.1 vs. 62.9 ± 11.1), total fruit (4.4 ± 1.1 vs. 3.7 ± 1.5), whole fruit (4.6 ± 1.0 vs. 4.2 ± 1.4), dairy (6.2 ± 2.7 vs. 4.8 ± 2.4), and empty calories (14.3 ± 3.2 vs. 11.1 ± 4.5) (*P* ≤ 0.05). Average daily intakes of foods and food groups that decreased included total dairy (*P* < 0.01), milk (*P* < 0.01), and non-juice fruit (*P* = 0.03). Dietary intake of calcium (*P* = 0.05) and vitamin D (*P* = 0.03) decreased. PTH increased from baseline (3.4 ± 1.6 vs. 3.8 ± 1.4 pmol/L, *P* = 0.04), while there was no change in 25-OH vitamin D. Ferritin decreased (385 ± 173 vs. 354 ± 161 pmol/L, *P* = 0.03) and soluble transferrin receptor increased (16.3 ± 3.7 vs. 17.1 ± 3.5 nmol/L, *P* = 0.01). There were no changes in glucose or lipids. Post-deployment, serum 25-OH vitamin D was inversely associated with PTH (*r* = −0.43, *P* < 0.01).

**Conclusions:**

HEI-2010 scores and dietary intake of milk, calcium, and vitamin D decreased following deployment. Serum PTH increased and iron stores were degraded. No Soldiers were iron deficient. Personnel that deploy frequently should maintain a high diet quality in the U.S. and while deployed by avoiding empty calories and consuming fruits, vegetables, and adequate sources of calcium, vitamin D, and iron. Improving availability and quality of perishable food during deployment may improve diet quality.

## Background

United States (U.S.) military personnel have deployed to support continuous military operations conducted since 2001. U.S. Army Special Operations Forces (SOF) Soldiers, in particular, deploy frequently [1] and are responsible for executing critical defense capabilities regarded as *special warfare*, which includes training, advising, and assisting host nations, as well as *surgical strike*, which includes some operations with humanitarian objectives such as hostage rescue [2]. SOF Soldiers must maintain high levels of physical and cognitive performance to respond effectively to mission requirements of ongoing and evolving conflicts [2]. Conventional and SOF Soldiers commonly conduct missions from overseas bases known as forward operating bases (FOB), which have evolved over time to provide considerable shelter and nourishment. However, the environment remains austere relative to living conditions in the U.S. and perishable and non-perishable food items can be difficult to transport to FOBs. The possibility of limited availability of food items requiring transport has the potential to decrease quality of dietary intake in deployed environments.

It is well established that optimal nutrition enhances physical performance and aids recovery [3]. In addition to consuming adequate calories and macronutrients to meet energy needs, specific micronutrients may also affect Soldier performance, particularly under conditions of increased physical activity. Iron status has been shown to decline in male and female recruits during basic training [4] and depleted iron stores impair performance by reducing oxygen transport capacity. Calcium and vitamin D are essential micronutrients to the structural integrity of bone. Furthermore, strenuous physical activity may increase release of parathyroid hormone (PTH) [5], a regulator of calcium metabolism which increases bone resorption and can decrease bone density. Suboptimal bone density and strength could increase risk of skeletal injury from both combat trauma and overuse (stress fracture), which may limit Soldiers’ abilities to fulfill deployment commitments. Additionally, vitamin D status has been investigated in relation to optimizing other aspects of physical performance, such as maximal oxygen consumption, muscle protein synthesis, and neuromuscular coordination [6]. Few studies have evaluated dietary intake in deployed environments [7, 8], therefore the objective of this study was to describe the effect of combat deployment on changes in diet quality and markers of nutritional status, including iron, vitamin D and PTH, glucose, and lipids of elite U.S. Army SOF Soldiers deployed to relatively austere environments.

## Methods

### Participants

**Fig. 1:**
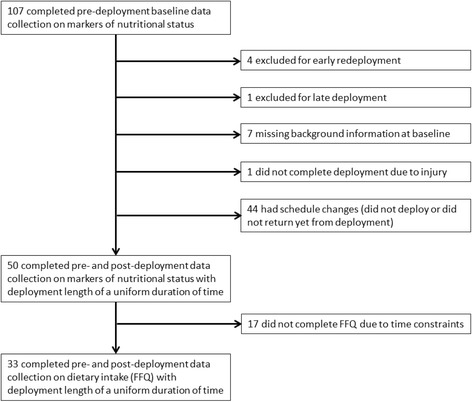
Flow chart of participant selection. Abbreviations: FFQ = food frequency questionnaire

Participants included in this study were healthy, active-duty Soldiers assigned to an elite U.S. Army Special Operations unit with full medical clearance, indicating they were free of disease or health conditions that would incur duty limitations or restrictions. Exclusion criteria included being deployed to a combat zone for ≥30 days within the previous four months, although no potential participants met this criterion for exclusion. All combat, combat support, and combat service support Soldiers assigned to the unit and eligible to deploy were invited to attend an informational study briefing. The briefing time was coordinated with unit leadership and scheduled to maximize the number of available Soldiers not otherwise actively engaged in training. Participants provided verbal informed consent after being recruited from the informational briefing with an ombudsman present. Approximately 90% of those that were briefed participated in the study. Pre-deployment baseline data on markers of nutritional status were collected between January and October of 2013 from 107 male participants 4–8 weeks before deployment. After excluding for early redeployment (*n* = 4), late deployment (*n* = 1), and missing background information at baseline (*n* = 7), post-deployment follow-up data on markers of nutritional status were collected from 50 participants during the reintegration period, within 10 days upon returning from deployment. The majority had measures obtained between 4 and 7 days (76%, 38/50) and fewer had measures obtained between 2 and 3 days (14%, 7/50) or later at 10 days (10%, 5/50). Loss to follow-up of the remaining participants occurred predominately due to changes in deployment schedules, such as that the participant did not deploy or did not return yet from deployment. One participant sustained a combat related injury and did not complete the deployment. The changing and often unpredictable nature of Soldier schedules and personnel assignments is common before, during, and after operational deployment and a similar pattern of loss to follow-up has been reported in previous deployment studies [7, 9]. Of the 50 participants with markers of nutritional status at baseline and post-deployment, all participants completed a food frequency questionnaire (FFQ) and background questionnaire at baseline and 33 also completed a FFQ and follow-up questionnaire upon return from deployment. Not all Soldiers were able to complete the FFQ and follow-up questionnaire upon return from deployment due to schedule time constraints during the reintegration period. A flow chart describing participant selection is provided in Fig. 1.

Deployment length was a uniform duration of time between 3 to 6 months (all Soldiers completed the same length of deployment). Eighty-six percent were deployed to Afghanistan and the remaining participants were deployed to other locations outside the continental U.S. All Soldiers had access to hot meals through cafeteria-style dining facilities for up to three meals per day, depending on individual schedules. Some Soldiers may have been required to be on duty during hours when dining facilities were closed, which occasionally (but not regularly) could have limited the number of meals they were able to obtain. Other shelf-stable food items were available to Soldiers through personal shipment, such as care packages, or supply by the unit. Meal, Ready-to-Eat (MRE) field rations were available if required by mission conditions. This study was approved by the Human Use Review Committee at the U.S. Army Research Institute of Environmental Medicine, Natick, MA. Investigators adhered to U.S. Army Regulation 70–25 and U.S. Army Medical Research and Material Command regulation 70–25 on the use of volunteers in research.

### Dietary intake assessment and demographic characteristics

Dietary intake over the previous 3-months was assessed at baseline and upon return from deployment with a 101-item self-administered paper and pencil version of the 2005 Block FFQ (NutritionQuest, Berkeley, CA) [10, 11]. The FFQ was chosen as the dietary assessment method to reduce participant burden and because the potential for research efforts to distract from military operations precluded the use of other dietary assessment methods during the deployment. Healthy Eating Index-2010 (HEI-2010) scores were calculated according to maximum and minimum score standards for each component, which have been described in detail elsewhere [12]. HEI-2010 is a measure of diet quality that assesses conformance with federal dietary guidelines [13–15]. The index is comprised of 12 components; 9 components assess compliance with adequate intakes (total vegetables, greens and beans, total fruit, whole fruit, whole grains, dairy, total protein foods, seafood and plant protein, and fatty acid ratio) and 3 components assess compliance with moderation (sodium, refined grains, and empty calories) [16]. Higher scores for each component are reflective of greater compliance with federal guidelines. The sum of each component score together yields the total HEI-2010 score, which ranges from 0 to 100.

Daily consumption of foods and food groups, dietary intake of key micronutrients, as well as carbohydrate, fat, and protein (expressed in grams and percent of calories), total caloric intake, and percent of calories from sweets and desserts were derived from the frequency and quantity of reported food items on the FFQ and corresponding nutrient values provided in the USDA’s Food and Nutrient Databases [17], as calculated by NutritionQuest. Self-reported frequency of intake of food items was assessed by asking participants to select “How often in the past 3 months” each food item listed on the FFQ was consumed. Response options included: ‘never’, ‘once per month’, ‘2–3 times per month’, ‘once per week’, ‘2 times per week’, ‘3–4 times per week’, ‘5–6 times per week’, or ‘every day’. Quantity of reported food items was then assessed by asking participants to select “How much on those days”. Response options included portion sizes appropriate for each food item and respondents were provided with standardized portion size pictures.

Demographic information (age, marital status, education level, combat classification, grade, prior combat deployments, and smoking status) was determined from a standardized self-administered demographic/background questionnaire at baseline. MRE field ration use was determined from a follow-up questionnaire administered upon return from deployment. Participants were asked: “During the past 6 months, during the time when you were deployed only, did you consume any MRE (Meal, Ready-to-Eat) type of product listed below? If so, how often did you use these products?” Products included ‘Meal, Ready-to-Eat’, ‘First Strike Ration’, ‘First Strike Bar’, and ‘Ergo Drink’ and response options of frequency of use included ‘never’, ‘once a month’, ‘once a week’, ‘2–6 times per week’, ‘once a day’, ‘2 times per day’, or ‘3+ times per day’.

### Markers of nutritional status

Blood samples were collected by median cubital venipuncture following an overnight fast at baseline and post-deployment. Immediately after collection, heparinized whole blood was used to measure hemoglobin and glucose using the iSTAT 1 handheld blood analyzer (Abbott Point of Care Inc., Princeton, NJ). Remaining samples were centrifuged, processed to isolate serum, frozen, and stored at −80 °C until the time of assay. Ferritin and PTH were measured using immunoassay with the Immulite 2000 XPi automated analyzer (Siemens Medical Solutions, Malvern, PA). Iron, total iron binding capacity (TIBC), total cholesterol, high density lipoprotein (HDL), low density lipoprotein (LDL), and triglycerides were assessed using a UniCel DxC 600 PRO clinical chemistry analyzer (Beckman Coulter, Brea, CA). Enzyme-linked immunosorbent assays (ELISA) were used to measure hepcidin (DRG Hepcidin ELISA, DRG International Inc., Springfield, NJ) and soluble transferrin receptor (sTfR) (Quantikine IVD Human Soluble Transferrin Receptor ELISA, R&D Systems Inc., Minneapolis, MN). Transferrin saturation (TS) was calculated as iron concentration × 100 / TIBC concentration. Levels of 25-hydroxy vitamin D (25-OH vitamin D) were assessed using radioimmunoassay (RIA; 25-Hydroxyvitamin D ^125^I RIA kit, DiaSorin Inc., Stillwater, MN).

### Statistical analysis

SAS statistical software (version 9.3; SAS Institute, Cary, NC) was used to perform all analyses. Findings were considered statistically significant for all analyses at *P* ≤ 0.05. Change in means from baseline to post-deployment for HEI-2010 scores, foods and food group intakes, nutrient intakes, and markers of nutritional status were assessed with a paired t-test. Paired measurements are well established to increase power. The proportion with iron status markers below threshold cut-offs indicative of iron deficiency anemia (ferritin <12 ng/mL and hemoglobin <13.7 g/dL) was also determined.

Decreases in dietary calcium and vitamin D intake and increases in serum PTH over the deployment were concurrently observed, therefore post hoc correlation and regression analyses were performed to determine associations with post-deployment PTH. PTH concentrations were regressed onto post-deployment serum 25-OH vitamin D concentrations, as well as total (dietary + supplemental) vitamin D and calcium intake during deployment using the General Linear Model (GLM) procedure in SAS. One outlier (where serum 25-OH vitamin D > 200 nmol/L) was omitted prior to analysis. Models were not adjusted for demographics or overall diet quality (HEI-2010 total score) during deployment because there were no univariate associations with PTH (*P* > 0.05).

## Results

### Demographic characteristics

Demographic characteristics of the deployed sample have been previously described [1]. On average, Soldiers were 33.5 ± 4.5 years old and completed 6.9 ± 3.9 prior combat deployments. The majority of Soldiers were married (38/50, 76%) and non-smokers (47/50, 94%). Thirty percent completed a bachelor degree (15/50), 52% completed an associate degree or some college (26/50), and 9% completed high school as the highest level of education attained. Seventy-eight percent were combat Soldiers (39/50), while 22% were combat support or combat service support Soldiers (11/50). Eighty-six percent were non-commissioned officers, between the pay grades of E5 to E-9 (43/50), and the remaining 14% were commissioned officers, between the pay grades of O4 to O5 (7/50).

### Change in dietary intake

HEI-2010 scores for total fruit, whole fruit, dairy, empty calories, and total HEI-2010 significantly decreased from baseline (Table 1). There were no changes in HEI-2010 scores for total vegetables, greens and beans, whole grains, total protein foods, seafood and plant protein, fatty acid ratio, sodium, or refined grains (*P* > 0.05). There were no significant differences in diet quality between the baseline sample and those with dietary intake data at follow-up (baseline total HEI-2010: 68.8 ± 8.5 vs. 70.3 ± 9.1, *P* > 0.05).

**Table 1:** Change in Healthy Eating Index-2010 scores^a^ during combat deployment among elite U.S. Army Special Operations soldiers (mean ± SD)

	*n* = 33				
	Max Score	Baseline	Deployed	Change	P
Total Vegetables	5	4.4 ± 0.7	4.3 ± 1.0	−0.1 ± 0.8	0.49
Greens and Beans^b^	5	4.6 ± 0.8	4.0 ± 1.5	−0.6 ± 1.7	0.06
Total Fruit^c^	5	4.4 ± 1.1	3.7 ± 1.5	−0.7 ± 1.4	<0.01
Whole Fruit^d^	5	4.6 ± 1.0	4.2 ± 1.4	−0.4 ± 1.2	0.05
Whole Grains	10	3.4 ± 2.7	3.8 ± 2.7	0.4 ± 2.2	0.29
Dairy	10	6.2 ± 2.7	4.8 ± 2.4	−1.4 ± 2.5	<0.01
Total Protein Foods	5	5.0 ± 0.2	4.9 ± 0.4	0.0 ± 0.4	0.68
Seafood and Plant Protein^e^	5	4.2 ± 1.0	3.8 ± 1.5	−0.4 ± 1.5	0.15
Fatty Acid Ratio^f^	10	6.5 ± 2.4	5.9 ± 2.9	−0.6 ± 3.1	0.27
Sodium	10	3.2 ± 2.2	2.7 ± 2.7	−0.4 ± 2.8	0.38
Refined Grains	10	9.6 ± 0.9	9.7 ± 0.9	0.0 ± 1.2	0.85
Empty Calories^g^	20	14.3 ± 3.2	11.1 ± 4.5	−3.2 ± 4.0	<0.01
Total Score	100	70.3 ± 9.1	62.9 ± 11.1	−7.4 ± 8.5	<0.01

^a^Scores calculated according to maximum (max) and minimum (min) score standards^9^

^b^Includes dark green vegetables, as well as beans and peas not included in total protein foods

^c^Includes fruit juice

^d^Excludes fruit juice

^e^Includes seafood, nuts, seeds, soy (other than beverages), and beans and peas not counted in total protein foods

^f^Polyunsaturated + monounsaturated / saturated fatty acids

^g^Percent of calories from solid fats, added sugars, and excess alcohol

Consumption of foods and food groups (per day average) that decreased from baseline included total dairy 1.78 ± 1.22 vs. 1.20 ± 0.78 cup-equivalents, *P* < 0.01), milk (1.10 ± 1.02 vs. 0.67 ± 0.62 cups, *P* < 0.01), and total non-juice fruit (1.28 ± 0.63 vs. 1.01 ± 0.64 cups, *P* = 0.03). There were no changes in consumption of total grains, whole grains, total vegetables, dark green vegetables, meat, fish, and poultry, eggs, nuts and seeds, legumes, yogurt, or cheese (*P* > 0.05).

Dietary intake of key nutrients (per day) that decreased from baseline included calcium (959 ± 432 vs. 831 ± 394 mg, *P* = 0.05) and vitamin D (184 ± 113 vs. 147 ± 107 IU, *P* = 0.03). There were no changes in dietary intakes of iron, zinc, sodium, magnesium, vitamin A, beta-carotene, vitamin B12, vitamin C, long-chain omega-3 fatty acids, fiber, total calories, or carbohydrate, fat, or protein expressed in grams or percent of calories (*P* > 0.05). The percent of calories from sweets and desserts slightly increased from before to during deployment (9.6 ± 5.1% vs. 13.4 ± 9.1%, *P* < 0.01).

### Change in markers of nutritional status

PTH significantly increased from baseline, while there was no change in 25-OH vitamin D (Table 2). Ferritin significantly decreased from baseline and sTfR significantly increased. There was no change in TS or hepcidin (*P* > 0.05). No participants met criteria for iron deficiency anemia at either baseline or post-deployment. There were no changes in glucose, total cholesterol, HDL, LDL, or triglycerides (*P* > 0.05).

**Table 2:** Change in markers of nutritional status following combat deployment among elite U.S. Army Special Operations soldiers (mean ± SD)

	*n* = 50			
	Baseline	Post-Deployment	Change	P
Vitamin D Status				
25-OH Vitamin D^a^ (nmol/L)	87.3 ± 25.8	89.3 ± 26.5	2.0 ± 23.4	0.55
Parathyroid Hormone (pmol/L)	3.4 ± 1.6	3.8 ± 1.4	0.4 ± 1.4	0.04
Iron Status				
Ferritin (pmol/L)	385 ± 173	354 ± 161	−31.0 ± 101	0.03
Soluble Transferrin Receptor (nmol/L)	16.3 ± 3.7	17.1 ± 3.5	0.8 ± 2.2	0.01
Transferrin Saturation (%)	39 ± 14	40 ± 14	1 ± 15	0.78
Hepcidin (nmol/L)	6.4 ± 5.4	6.0 ± 3.8	−0.4 ± 4.9	0.61
Glucose and Lipid Status				
Glucose (mmol/L)	5.06 ± 0.35	5.07 ± 0.30	0.00 ± 0.40	0.94
Total Cholesterol (mmol/L)	5.00 ± 0.99	5.01 ± 0.90	0.01 ± 0.62	0.89
HDL^a^ (mmol/L)	1.47 ± 0.30	1.43 ± 0.29	−0.03 ± 0.18	0.23
LDL^a^ (mmol/L)	3.07 ± 0.84	3.13 ± 0.72	0.07 ± 0.54	0.40
Triglycerides (mmol/L)	1.01 ± 0.50	0.96 ± 0.57	−0.05 ± 0.46	0.46

^a^ *Abbreviations*: *25-OH Vitamin D* 25-hydroxy vitamin D, *HDL* high density lipoprotein, *LDL* low density lipoprotein

**Fig. 2:**
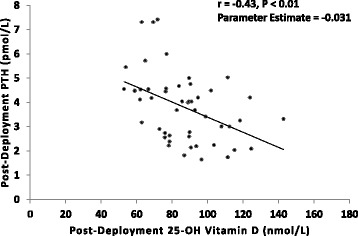
Association between post-deployment serum 25-OH vitamin D and PTH concentrations among U.S. Army SOF Soldiers. Abbreviations: SOF = Special Operations Forces; PTH = parathyroid hormone; 25-OH vitamin D = 25-hydroxy vitamin D

Post-deployment serum 25-OH vitamin D was inversely associated with post-deployment PTH (Fig. 2). Total vitamin D intake during deployment was also inversely associated with post-deployment PTH (*r* = −0.35, parameter estimate = −0.002, *P* = 0.05). The association between total calcium intake during deployment and post-deployment PTH was also in the inverse direction (*r* = −0.30, parameter estimate = −0.001, *P* = 0.09), but did not achieve significance.

### Use of field rations

The proportion reporting use of MRE field rations during deployment was 3/33 (once per month), 2/33 (once per week), and 2/33 (2–6 times per week). First Strike ration use was reported by 2/33 (once per month) and 1/33 (once per week), First Strike bar use was reported by 2/33 (once per month) and 1/33 (once per week), and Ergo drink use was reported by 1/33 (once per month).

### Location differences

Results were not stratified by location because too few participants were deployed to locations other than Afghanistan and there were no significant differences in important outcome measures according to location of Afghanistan vs. other (change in total HEI-2010: -7.6 ± 8.9 vs. -5.2 ± 2.9, change in 25-OH vitamin D: 1.9 ± 23.8 vs. 2.6 ± 22.6 nmol/L, change in PTH: 3.4 ± 12.6 vs. 6.5 ± 15.5 ng/L, change in ferritin: −23.4 ± 104.9 vs. -78.2 ± 59.2 pmol/L, and change in sTfR: 0.8 ± 2.4 vs. 1.0 ± 0.8 nmol/L, *P* > 0.05).

## Discussion

A decrease in diet quality as measured by total HEI-2010 score was observed during deployment. Specific HEI-2010 component scores that decreased from baseline included total fruit, whole fruit, dairy, and empty calories. To our knowledge, this is the first study to describe changes in patterns of diet quality induced by military deployment. By contrast, in a training environment, increases in diet quality as measured by HEI scores were observed among military recruits in basic combat training who entered training with lower diet quality [18]. The average baseline HEI score (70.3 ± 9.1) of SOF Soldiers in the present study most closely approximates the average baseline HEI score (73.1 ± 6.2) of recruits in the highest tertile of diet quality in basic combat training [18]. This is relative to average HEI scores reported for recruits in the middle tertile (60.3 ± 3.4) and lowest tertile (46.9 ± 4.4) [18]. Because SOF Soldiers must undergo rigorous assessment and selection procedures and demonstrate competency during training exercises to obtain their position [2], the high diet quality observed at baseline may be indicative of dietary patterns that experienced SOF Soldiers adopt to optimize performance and meet training and mission requirements. Soldiers with relatively higher diet quality prior to deployment may be expected to experience a reduction in diet quality, possibly related to limitations in the availability and/or quality (taste) of perishable food items available in dining facilities on overseas FOBs.

Changes in intake of specific foods and food groups were consistent with HEI-2010 scores; total non-juice fruit, total dairy, and milk decreased. The decreases in dairy intake were attributable to decreases in milk intake because yogurt and cheese intake did not change. Decreased milk intake was observed concurrently with decreased calcium and vitamin D intake and increased serum PTH. While decreased serum 25-OH vitamin D was not observed, sun exposure during deployment to Iraq and Afghanistan has been previously reported [19] and endogenous vitamin D synthesis from sun exposure may have offset decreases that may have occurred from reduced dietary intake of vitamin D. Nonetheless, post-deployment serum 25-OH vitamin D and total vitamin D intake during deployment were both inversely associated with post-deployment PTH. The observed increase in serum PTH likely indicates lowered serum ionized calcium concentrations since PTH synthesis increases to restore calcium homeostasis by increasing intestinal calcium absorption, kidney calcium resorption, and bone resorption to release calcium into the bloodstream [20]. PTH may also increase as a result of strenuous physical activity [5] and SOF personnel report increasing the time spent engaged in voluntary cardiovascular and strength training exercise during deployment [1]. It is not known whether increased PTH induced alterations to measures of bone density or strength in this study. However, conditions during deployment, such as decreased calcium and vitamin D intake coupled with increased physical activity, which promote increased bone resorption and may not be offset by bone formation, have the potential to compromise bone integrity and increase skeletal injury risk due to both traumatic force and micro-traumatic repetitive overuse. Musculoskeletal injuries account for the majority of combat wounds [21] with fracture accounting for 26% of extremity combat wounds [22]. Non-combat musculoskeletal injuries are also the most common reason for medical evacuation during deployment [23].

The reasons for decreased milk intake (and resulting decreases in dietary calcium and vitamin D intake) during deployment are unknown. However, if related to availability and/or poor quality, improving availability and/or taste of milk products made available to Soldiers during deployment may aid in maintaining adequate calcium and vitamin D intakes. When this is not possible due to logistical issues related to the acquisition and maintenance of quality perishable food items, supplemental calcium and vitamin D should be considered for further study. For example, supplemental calcium and vitamin D have been shown to maintain PTH and improve measures of bone mineral density and bone mineral content among male and female recruits during basic combat training [24].

Decreases in ferritin and corresponding increases in sTfR over the deployment were also observed. Ferritin is the protein that stores iron and sTfR is the extracellular component of the transferrin receptor, which binds the iron-transferrin complex for transport and delivery of iron into cells; sTfR increases in response to decreased iron stores. Despite alterations to these iron status markers, iron status was robust in this sample of male Soldiers, and neither iron deficiency nor iron deficiency anemia was apparent in the study population. Alterations to iron status markers are noteworthy, however, because impaired iron status has the potential to degrade many aspects of Soldier performance during deployment. Iron is present in the brain and iron supplementation may improve cognitive performance among those with iron-deficiency anemia [25]. Adequate iron status is recognized as important to immune function [26] and therefore resistance to infection, as well as temperature regulation [27]. Because iron is required for oxygen transport, the role of iron-deficiency anemia in degrading physical performance, including aerobic capacity and physical work capacity, are well documented [28]. Due to the decline in iron stores noted in this study, maintaining iron status could be a concern for Soldiers experiencing repeated deployment, although the effects of repeated deployment on nutritional status have not been explored.

Similar to our study, a previous study of male Royal Marines also observed decreases in ferritin following 3- and 6-months of deployment to Afghanistan [7]. The consistency of these findings suggests conditions common during deployment may contribute to the degradation of iron stores. Increased physical activity, which may arise either from mission requirements, voluntary physical activity, or both, may be a contributing factor. Military training characterized by sustained submaximal exercise has been previously shown to degrade iron status in males [4, 29] and females [4]. Female military recruits, in particular, may be at higher risk of iron deficiency anemia [30, 31] and iron supplementation of female recruits during basic combat training resulted in attenuation of degraded iron status [32, 33] and faster run times in anemic recruits [32]. Because poor iron status may resolve with supplementation, future studies may therefore aim to assess the effect of deployment on iron status of physically active female military personnel.

No changes in dietary intake of calories, or carbohydrate, fat, or protein expressed in grams or percent of calories were observed. This is consistent with one previous study of a 12-month deployment to Iraq among combat support Soldiers which found no changes in dietary intake of calories or macronutrients (grams) assessed by FFQ [8]. The study of Royal Marines also observed no changes in percentages of macronutrient intake [7]. However, in the present study, the HEI-2010 component score for empty calories decreased, indicating an increase in percentage of calories consumed from empty calorie sources, such as solid fats and added sugars. A small increase in percentage of calories from sweets and desserts was observed (9.6 ± 5.1% vs. 13.4 ± 9.1%). Further analyses indicate that the proportion of participants reporting certain sweets and dessert food sources ≥ once per week increased from before to during deployment: 7/33 vs. 15/33 for cookies, 6/33 vs. 12/33 for chocolate candy, and 0/33 vs. 6/33 for regular candy. In addition, MRE field rations were used infrequently. This suggests that caloric intake from food sources other than field rations was not limited for the majority of Soldiers during deployment. Furthermore, energy restriction due to inadequate caloric intake is unlikely to have occurred because as we previously reported, the majority of Soldiers did not lose weight during the deployment, and those that did lose weight engaged in high levels of voluntary physical activity [1].

A limitation of this study is that participants were elite SOF Soldiers with relatively high levels of diet quality at baseline, as indicated by HEI-2010 scores. These findings may not be generalizable to Soldiers with lower diet quality at baseline because decreases in diet quality occurring over deployment may not be as pronounced. Despite this limitation, SOF Soldiers deploy frequently and results are applicable to these Soldiers, who are likely to deploy again in the future. Another limitation is that post-deployment follow-up data on markers of nutritional status was collected during the reintegration period and markers could have changed during this time. However, this may not be a valid limitation because our finding of a decrease in serum ferritin replicates the same finding observed in the previous study of male Royal Marines, in which data were collected during deployment [7]. It should also be noted that the overall sample size of this study was small, which is reflective of the small population of elite SOF Soldiers relative to the population of conventional Soldiers in the U.S. Army. Larger studies with more participants may be required to detect smaller effects than those observed in the present study.

## Conclusions

Overall, a decrease in diet quality (HEI-2010) and decrease in intake of foods and food groups (total non-juice fruit, total dairy, and milk) were observed during operational deployment. Decreased dietary intake of calcium and vitamin D, as well as increased serum PTH were concurrently observed. Iron stores were affected, as ferritin decreased and sTfR increased. These findings are important because the condition of deployment is chronic for SOF Soldiers that deploy frequently. Military personnel that deploy frequently should continue to be encouraged to consume fruits and vegetables, as well as adequate food sources of calcium and vitamin D (such as dairy products) and iron (such as meat and poultry) when in the U.S. and during deployment to attenuate potential declines in nutritional status.

Improving the availability and quality of perishable food offered during deployment may prevent observed declines in diet quality. However, it should be recognized that SOF Soldiers may be required to deploy without advanced notice to exceedingly austere environments that do not have the existing infrastructures to provide nourishment commonly available at major overseas FOBs. In these instances, optimizing nutritional status prior to deployment is critical and studies examining alternatives to obtaining adequate intakes of important nutrients, such as calcium, vitamin D, and iron, in austere environments should be examined by conducting randomized controlled trials. Training environments that mimic the austere conditions of deployment and limit access to perishable food items may be ideally suited for conducting trials that assess the safety and efficacy of these alternatives, which may include dietary supplements, meal replacements, or other food products. Future observational studies should also be conducted and include measures of bone density or strength and populations at higher risk for poor iron status, such as physically active female military personnel. Observational studies should also assess how host factors, such as baseline diet quality and physical activity levels during deployment, in addition to environmental factors, such as sun exposure and access to perishable food items, may differentially affect changes occurring over deployment.

## Abbreviations

25-OH vitamin D: 25-hydroxy vitamin D
ELISA: enzyme-linked immunosorbent assay
FFQ: food frequency questionnaire
FOB: forward operating base
HDL: high density lipoprotein
HEI-2010: healthy eating index-2010
LDL: low density lipoprotein
MRE: Meal, Ready-to-Eat
PTH: parathyroid hormone
SOF: special operations forces
sTfR: soluble transferrin receptor
TIBC: total iron binding capacity
TS: transferrin saturation
US: United States

## References

[1] Farina EK, Taylor JC, Means GE, Williams KE, Murphy NE, Margolis LM, Pasiakos SM, Lieberman HR, McClung JP (2017). Effects of combat deployment on anthropometrics and physiological status of U.S. Army special operations forces soldiers. Mil Med.

[2] Special Operations, Army doctrine publication 3–05. Washington, DC: Headquarters, Department of the Army; 2012.

[3] Joint Position Statement: nutrition and athletic performance. American College of Sports Medicine, American dietetic association, and dietitians of Canada. Med Sci Sports Exerc 2000;32(12):2130-2145. 10.1097/00005768-200012000-00025 11128862

[4] Yanovich R, Karl JP, Yanovich E, Lutz LJ, Williams KW, Cable SJ, et al. Effects of basic combat training on iron status in male and female soldiers: a comparative study. US Army Med Dep J. 2015:67–73. 26101908

[5] Bouassida A, Latiri I, Bouassida S, Zalleg D, Zaouali M, Feki Y, Gharbi N, Zbidi A, Tabka Z (2006). Parathyroid hormone and physical exercise: a brief review. J Sports Sci Med.

[6] Moran DS, McClung JP, Kohen T, Lieberman HR (2013). Vitamin d and physical performance. Sports Med.

[7] Fallowfield JL, Delves SK, Hill NE, Cobley R, Brown P, Lanham-New SA, Frost G, Brett SJ, Murphy KG, Montain SJ, Nicholson C, Stacey M, Ardley C, Shaw A, Bentley C, Wilson DR, Allsopp AJ (2014). Energy expenditure, nutritional status, body composition and physical fitness of Royal Marines during a 6-month operational deployment in Afghanistan. Br J Nutr.

[8] Carlson AR, Smith MA, McCarthy MS. Diet, physical activity, and bone density in soldiers before and after deployment. US Army Med Dep J. 2013:25–30. 23584905

[9] Hill NE, Woods DR, Delves SK, Murphy KG, Davison AS, Brett SJ, Quinton R, Turner S, Stacey M, Allsopp AJ, Fallowfield JL (2015). The gonadotrophic response of Royal Marines during an operational deployment in Afghanistan. Andrology.

[10] Block G, Woods M, Potosky A, Clifford C (1990). Validation of a self-administered diet history questionnaire using multiple diet records. J Clin Epidemiol.

[11] Mares-Perlman JA, Klein BE, Klein R, Ritter LL, Fisher MR, Freudenheim JL (1993). A diet history questionnaire ranks nutrient intakes in middle-aged and older men and women similarly to multiple food records. J Nutr.

[12] Guenther PM, Casavale KO, Reedy J, Kirkpatrick SI, Hiza HA, Kuczynski KJ, Kahle LL, Krebs-Smith SM (2013). Update of the healthy eating index: HEI-2010. J Acad Nutr Diet.

[13] Kennedy ET, Ohls J, Carlson S, Fleming K (1995). The healthy eating index: design and applications. J Am Diet Assoc.

[14] Guenther PM, Reedy J, Krebs-Smith SM (2008). Development of the healthy eating index-2005. J Am Diet Assoc.

[15] Guenther PM, Reedy J, Krebs-Smith SM, Reeve BB (2008). Evaluation of the healthy eating index-2005. J Am Diet Assoc.

[16] Guenther PM, Kirkpatrick SI, Reedy J, Krebs-Smith SM, Buckman DW, Dodd KW, Casavale KO, Carroll RJ (2014). The healthy eating index-2010 is a valid and reliable measure of diet quality according to the 2010 dietary guidelines for Americans. J Nutr.

[17] Ahuja JK, Moshfegh AJ, Holden JM, Harris E (2013). USDA food and nutrient databases provide the infrastructure for food and nutrition research, policy, and practice. J Nutr.

[18] Lutz LJ, Gaffney-Stomberg E, Scisco JL, Cable SJ, Karl JP, Young AJ, et al. Assessment of dietary intake using the healthy eating index during military training. US Army Med Dep J. 2013:91–7. 24146246

[19] Powers JG, Patel NA, Powers EM, Mayer JE, Stricklin GP, Geller AC (2015). Skin cancer risk factors and preventative behaviors among United States military veterans deployed to Iraq and Afghanistan. J Invest Dermatol.

[20] Boden SD, Kaplan FS (1990). Calcium homeostasis. Orthop Clin North Am.

[21] Owens BD, Kragh JF, Wenke JC, Macaitis J, Wade CE, Holcomb JB (2008). Combat wounds in operation Iraqi freedom and operation enduring freedom. J Trauma.

[22] Owens BD, Kragh JF, Macaitis J, Svoboda SJ, Wenke JC (2007). Characterization of extremity wounds in operation Iraqi freedom and operation enduring freedom. J Orthop Trauma.

[23] Cohen SP, Brown C, Kurihara C, Plunkett A, Nguyen C, Strassels SA (2010). Diagnoses and factors associated with medical evacuation and return to duty for service members participating in operation Iraqi freedom or operation enduring freedom: a prospective cohort study. Lancet.

[24] Gaffney-Stomberg E, Lutz LJ, Rood JC, Cable SJ, Pasiakos SM, Young AJ, McClung JP (2014). Calcium and vitamin D supplementation maintains parathyroid hormone and improves bone density during initial military training: a randomized, double-blind, placebo controlled trial. Bone.

[25] Falkingham M, Abdelhamid A, Curtis P, Fairweather-Tait S, Dye L, Hooper L (2010). The effects of oral iron supplementation on cognition in older children and adults: a systematic review and meta-analysis. Nutr J.

[26] Ekiz C, Agaoglu L, Karakas Z, Gurel N, Yalcin I (2005). The effect of iron deficiency anemia on the function of the immune system. Hematol J.

[27] Brigham D, Beard J (1996). Iron and thermoregulation: a review. Crit Rev Food Sci Nutr.

[28] Haas JD, Brownlie T (2001). Iron deficiency and reduced work capacity: a critical review of the research to determine a causal relationship. J Nutr.

[29] Radomski MW, Sabiston BH, Isoard P (1980). Development of “sports anemia” in physically fit men after daily sustained submaximal exercise. Aviat Space Environ Med.

[30] McClung JP, Marchitelli LJ, Friedl KE, Young AJ (2006). Prevalence of iron deficiency and iron deficiency anemia among three populations of female military personnel in the US Army. J Am Coll Nutr.

[31] Myhre KE, Webber BJ, Cropper TL, Tchandja JN, Ahrendt DM, Dillon CA, Haas RW, Guy SL, Pawlak MT, Federinko SP (2015). Prevalence and impact of anemia on basic trainees in the US air force. Sports Med Open.

[32] McClung JP, Karl JP, Cable SJ, Williams KW, Nindl BC, Young AJ, Lieberman HR (2009). Randomized, double-blind, placebo-controlled trial of iron supplementation in female soldiers during military training: effects on iron status, physical performance, and mood. Am J Clin Nutr.

[33] Karl JP, Lieberman HR, Cable SJ, Williams KW, Young AJ, McClung JP (2010). Randomized, double-blind, placebo-controlled trial of an iron-fortified food product in female soldiers during military training: relations between iron status, serum hepcidin, and inflammation. Am J Clin Nutr.

